# Pre-Diabetes Augments Neuropeptide Y_1_- and α_1_-Receptor Control of Basal Hindlimb Vascular Tone in Young ZDF Rats

**DOI:** 10.1371/journal.pone.0046659

**Published:** 2012-10-05

**Authors:** Nicole M. Novielli, Baraa K. Al-Khazraji, Philip J. Medeiros, Daniel Goldman, Dwayne N. Jackson

**Affiliations:** 1 Department of Medical Biophysics, The University of Western Ontario, London, Ontario, Canada; 2 Biomedical Engineering Program, The University of Western Ontario, London, Ontario, Canada; Max-Delbrück Center for Molecular Medicine (MDC), Germany

## Abstract

**Background:**

Peripheral vascular disease in pre-diabetes may involve altered sympathetically-mediated vascular control. Thus, we investigated if pre-diabetes modifies baseline sympathetic Y_1_-receptor (Y_1_R) and α_1_-receptor (α_1_R) control of hindlimb blood flow (Q_fem_) and vascular conductance (VC).

**Methods:**

Q_fem_ and VC were measured in pre-diabetic ZDF rats (PD) and lean controls (CTRL) under infusion of BIBP3226 (Y_1_R antagonist), prazosin (α_1_R antagonist) and BIBP3226+prazosin. Neuropeptide Y (NPY) concentration and Y_1_R and α_1_R expression were determined from hindlimb skeletal muscle samples.

**Results:**

Baseline Q_fem_ and VC were similar between groups. Independent infusions of BIBP3226 and prazosin led to increases in Q_fem_ and VC in CTRL and PD, where responses were greater in PD (*p*<0.05). The percent change in VC following both drugs was also greater in PD compared to CTRL (*p*<0.05). As well, Q_fem_ and VC responses to combined blockade (BIBP3226+prazosin) were greater in PD compared to CTRL (*p*<0.05). Interestingly, an absence of synergistic effects was observed within groups, as the sum of the VC responses to independent drug infusions was similar to responses following combined blockade. Finally, white and red vastus skeletal muscle NPY concentration, Y_1_R expression and α_1_R expression were greater in PD compared to CTRL.

**Conclusions:**

For the first time, we report heightened baseline Y_1_R and α_1_R sympathetic control of Q_fem_ and VC in pre-diabetic ZDF rats. In support, our data suggest that augmented sympathetic ligand and receptor expression in pre-diabetes may contribute to vascular dysregulation.

## Introduction

In the peripheral vasculature, sympathetic neurons regulate arteriolar tone through the release of norepinephrine (NE) and neuropeptide Y (NPY). NE has been considered the primary neurotransmitter in maintenance of baseline arteriolar tone [1] through its interaction with alpha-adrenergic receptors (αR) located on vascular smooth muscle cells, causing vasoconstriction. NPY is co-stored and co-released with NE and acts on neuropeptide Y_1_ receptors (Y_1_R), to cause potent and prolonged vasoconstriction [2], [3], [4]. Interestingly, post-synaptic co-activation of Y_1_R and α_1_R by NPY and NE leads to synergistic vasoconstrictive effects [4]. Although recent evidence has shown that NPY contributes modestly to baseline vascular tone in skeletal muscle of male rats [5], its effects are suggested to predominate under conditions of elevated sympathetic nerve activity [6], [7], [8].

A large proportion of the body's resistance vasculature lies within skeletal muscle, which is highly regulated by sympathetic nerve activity (SNA) to maintain blood pressure and blood flow distribution under healthy conditions. However, in type 2 diabetes, sympathetic regulation of vascular tone can become augmented, leading to alterations in normal blood flow control. Type 2 diabetes is commonly associated with vascular disease, however recent findings indicate that cardiovascular complications may be initiated in the pre-diabetic state, before the diagnosis of type 2 diabetes [9], [10]. Pre-diabetes is characterized by the concomitant presence of hyperinsulinemia, impaired glucose tolerance and insulin resistance and occurs prior to overt pancreatic β-cell failure. Of note, hyperinsulinemia stimulates SNA and may play a role in autonomic and vascular dysfunction associated with the disease [11]. In humans, hyperinsulinemia is associated with elevated SNA and correlates with the degree of insulin resistance [12], [13], [14]. Moreover, systemic infusion of insulin in rats has been shown to preferentially increase lumbar SNA [15], [16], [17], [18]. Studies using animal models of pre-diabetes and the metabolic syndrome have reported augmented α-adrenergic vascular responsiveness to adrenergic agonists in isolated vascular preparations [19], [20]. As noted above, NPY-mediated vascular modulation becomes more pronounced under conditions of elevated SNA [6], [7], [8]; however, to date, studies addressing NPY/Y_1_R-mediated vascular control in pre-diabetes are lacking. Furthermore, there have been no studies investigating NPY and α-adrenergic co-modulation of vascular control in pre-diabetes.

The overall aim of the present study was to investigate if pre-diabetes modifies sympathetic Y_1_R and α_1_R control of basal skeletal muscle blood flow (Q_fem_) and vascular conductance (VC). Thus, we tested the independent and dependent (synergistic) functional contributions of endogenous Y_1_R and α_1_R activation on Q_fem_ and VC *in vivo* and hypothesized that pre-diabetes augments Y_1_R and α_1_R vascular modulation. Concurrently, we hypothesized that skeletal muscle NPY concentration and Y_1_R and α_1_R expression would be upregulated in pre-diabetic rats.

## Materials and Methods

All animal procedures were approved by the Council on Animal Care at The University of Western Ontario (protocol number: 2008-066). All invasive procedures were performed under α-chloralose and urethane anesthetic, and all efforts were made to minimize animal suffering.

### Animals

Nine seven-week-old male ZDF rats (PD) and 8 age-matched lean controls (CTRL) (Charles River Laboratories, Saint-Constant, Quebec, Canada) were used in this study. The inbred ZDF rat is affected by a homozygous mutation of the leptin receptor (fa/fa), therefore leptin is unable to suppress appetite [21]. When fed a high fat diet (i.e., Purina 5008 rat chow), these animals become obese, hyperinsulinemic, insulin resistant and hyperglycemic by 7 weeks of age [20], [21], characteristic of the pre-diabetic condition in humans [22], [23]. This phenotype is absent in the ZDF lean rats heterozygous for the leptin receptor mutation (fa/+), and thus served as the control group in this study. Animals were housed in animal care facilities in a temperature (24°C) and light (12-hour cycle)-controlled room, fed Purina 5008 rat chow (Ralston Purina, St. Louis, MO, USA) and allowed to eat and drink water *ad libitum*. Prior to surgery, animals were anesthetized with an intraperitoneal injection of α-chloralose (80 mg/kg) and urethane (500 mg/kg). This anesthetic was ideal for this study as it leaves autonomic, cardiovascular and respiratory function intact [24]. Internal body temperature was monitored via a rectal temperature probe and maintained at 37°C with the use of a thermally controlled water-perfused heating pad.

### Surgery

A mid-neck incision was made and a tracheal cannula was introduced to facilitate spontaneous respiration. End-tidal CO_2_ and O_2_ measures were made from expired air between pharmacological perturbations throughout the experiment using a breath-by-breath gas analyzer (ADInstruments, Colorado Springs, CO, USA). The left common carotid artery was cannulated (PE-50 tubing) to allow for recording of arterial blood pressure via the amplified signal of a pressure transducer using a PowerLab system (model ML118 PowerLab Quad Bridge Amplifier; model MLT0699 BP Transducer, ADInstruments, Colorado Springs, CO, USA). The right jugular vein was cannulated to maintain a constant infusion of anesthetic to the animal (α-chloralose: 8–16 mg/kg/hr, urethane: 50–100 mg/kg/hr).

Through a midline abdominal incision, gut contents were carefully moved aside within the abdominal cavity and covered with sterile gauze moistened with sterile saline (0.9% NaCl). Sterile cotton swabs were then used to expose the descending aorta and right iliac artery, isolating it from the right iliac vein and its surrounding fat. The right iliac artery was cannulated (PE-50 tubing) and the cannula was advanced to the bifurcation of the descending aorta. This cannula was used for localized drug delivery to the left hindlimb. Following cannulation, gauze was removed and care was taken to reposition the gut. Incisions were closed with sterile wound clips (9 mm stainless steel wound clips). A blood sample was then taken from the carotid cannula in order to evaluate blood glucose levels, lactate levels, and pH using an iSTAT portable clinical analyzer (Abbott Laboratories, Abbott Park, IL, USA).

Using microscopic assistance, the left femoral artery was carefully isolated from surrounding nerves and vessels. Q_fem_ was measured beat-by-beat using a Transonic flow probe (0.7 PSB) and flowmeter (model TS420 Perivascular Flowmeter Module; Transonic Systems, Ithica, NY, USA). The flow probe was placed around the left femoral artery ∼3 mm from the femoral triangle and innocuous water-soluble ultrasound gel was applied over the opened area of the left hindlimb to keep tissue hydrated and to maintain adequate flow signal.

### Experimental protocol

Once surgery was completed, animals recovered for 1 hour. Prior to drug treatments, vehicle (160 µl of 0.9% saline) was delivered, followed by a 15-minute recovery period. Baseline data were recorded for 5 minutes followed by five separate drug infusions [5], [25], [26], [27]. Using a repeated measures design, drug infusions were delivered at a rate of 16 µl/sec in the following order: 1) 250 µl of 0.2 µg/kg acetylcholine chloride (ACh, Sigma-Aldrich, St. Louis, MO, USA), 2) 160 µl of 100 µg/kg BIBP3226, a specific Y_1_R antagonist (TOCRIS, Ellisville, MO, USA), 3) 160 µl of 20 µg/kg prazosin, a specific α_1_R antagonist (Sigma-Aldrich, St. Louis, MO, USA), 4) combined 100 µg/kg BIBP3226+20 µg/kg prazosin, and 5) 160 µl of 5 µg/kg sodium nitroprusside (SNP, i.v., sodium nitroprussiate dihydrate, Sigma-Aldrich, St. Louis, MO, USA). Since the hemodynamic effects of prazosin are long lasting, BIBP3226 (Y_1_R antagonist) was administered first in all experiments. When hemodynamic variables returned to baseline (30–40 minutes), prazosin (α_1_R antagonist) was infused. Once responses to prazosin peaked and stabilized (∼5 minutes), combined blockade (Y_1_R+α_1_R antagonist) was achieved by a subsequent infusion of BIBP3226 (100 µg/kg). In a previous study (using a similar protocol), we addressed the effects of randomized versus fixed delivery of BIBP3226 and prazosin and reported no effect of randomization [27].

### Insulin immunoassay

Insulin levels were determined from plasma samples using an ELISA and by following manufacturer's instruction (ALPCO Immunoassays, Salem, NH, USA). All samples and standards (10 µl) were distributed in duplicate in the provided 96-well immunoplate. Seventy-five microliters of horseradish peroxidase (HRP)-labeled monoclonal anti-insulin antibody was added to each well and incubated at room temperature for 2 hours. The immunoplate was then washed 6 times with assay wash buffer. Following washing, 100 µl of tetramethylbenzidine (TMB) peroxidase substrate solution was added to each well and incubated for 15 minutes at room temperature. The reaction was then terminated with 100 µl of stop solution, and the optical absorbance of each well was read at 450 nm (Bio-Rad iMark Microplate Reader, Bio-Rad, Hercules, CA, USA).

### NPY immunoassay and Western blotting

Analyses were carried out on two different skeletal muscle groups known to contain differing expression of slow-twitch oxidative (SO), fast-twitch glycolytic (FG), and fast-twitch oxidative-glycolytic (FOG) fiber types. The use of skeletal muscle groups expressing differing ratios of fiber types was based on early work by others showing that blood flow to such muscles is distributed differently at rest [28] and during exercise [28], [29]. We chose to analyze vastus muscle, as it comprises the bulk of muscle tissue in the hindlimb and plays a major role in locomotion. With the animal under deep surgical anesthesia, skeletal muscle samples were taken from red vastus (RV; expressing FOG>FG>SO fibers) and white vastus (WV; expressing FG>FOG) [30], [31] and were flash-frozen in liquid nitrogen. Animals were euthanized after tissue harvesting by an overdose of anesthetic. The same muscle tissue samples were used in all assays (NPY immunoassay and Western blot).

NPY concentration was determined in whole muscle tissue homogenates (from white and red vastus; see below for preparation of homogenate and total protein determination) and standards (50 µl duplicate samples) using a competitive immunoassay (Bachem Bioscience, King of Prussia, PA, USA). All samples were incubated at room temperature for 2 hours. The immunoplate was then washed 5 times with 300 µl per well of assay buffer. Wells were incubated at room temperature with 100 µl of streptavidin-HRP for 1 hour. The immunoplate was washed again 5 times with 300 µl per well of assay buffer. Following washing, 100 µl of a TMB peroxidase substrate solution was added to all wells. After a 40 minute incubation at room temperature the reaction was terminated by the addition of 100 µl 2 N HCl. Finally, the optical absorbance of each well was read at 450 nm (Bio-Rad Ultramark Microplate Imaging System, Bio-Rad, Hercules, CA, USA). Absorbance measures were converted to NPY concentration by comparison with the 10-point standard curve. Results are given as a ratio of pg NPY (per µg tissue), relative to protein concentration, as computed from amount of total protein loaded per well. The assay has a minimum detectable concentration of 0.04–0.06 ng per ml or 2–3 pg per well (manufacturer's data).

White and red vastus skeletal muscle tissue was removed from the hindlimb and flash frozen in liquid nitrogen. Approximately 100 mg of tissue was cut from the whole muscle and homogenized in 2 mL of radioimmunoprecipitation assay lysis buffer (50 mM Tris-HCl pH 7.4, 150 mM NaCl, 1% IGEPAL, 1% Sodium deoxycholate, 0.1% SDS, 100 mM EDTA) containing protease inhibitor cocktail (104 mM AEBSF, 80 mM aprotinin, 2.1 mM leupeptin, 3.6 mM betastatin, 1.5 mM pepstatin A, 1.4 mM ME-64, Sigma-Aldrich, St. Louis, MO, USA). Samples were then centrifuged at 4°C for 25 minutes at 14000 rpm and supernatant was collected and then stored at −80°C until ready for use. Protein concentration was determined using the Bradford protein assay [32]. Fifty micrograms of protein from each sample was loaded on a 4% to 12% gradient gel and separated by SDS-PAGE. After electrophoresis, proteins were transferred at a constant voltage to polyvinylidene fluoride membranes. Membranes were blocked in 5% milk in tris-buffered saline+Tween20 (0.5%) (TTBS) at 4°C for 5 hours. Membranes were washed in TTBS and incubated overnight at 4°C in one of two primary antibodies in 5% milk in TTBS, specific to rat, human or mouse: 1) Y_1_R (rabbit polyclonal to NPY1R, Cat no. ab73897, Abcam, Cambridge, MA, USA), and 2) α_1_R (rabbit polyclonal to alpha 1 adrenergic receptor, Cat no. ab3462, Abcam, Cambridge, MA, USA). After incubation, membranes were washed in TTBS then incubated in secondary antibody conjugated to HRP (goat anti-rabbit IgG, Cat no. A0545, Sigma Aldrich, St Louis, MO, USA) in 5% milk in TTBS for 1 hour at room temperature. Membranes were washed and bands were detected using Immun-Star WesternC© chemiluminescent kit (Bio-Rad, Hercules, CA, USA) and imaged with a ChemiDoc XRS System (Bio-Rad, Hercules, CA, USA). Membranes were immediately washed, stripped, and blocked in 5% bovine serum albumin for 1 hour at room temperature. Membranes were washed and incubated in primary antibody specific to β-actin (loading control, anti-beta actin, rabbit polyclonal, Cat no. ab16039, Abcam, Cambridge, MA, USA) for 1 hour at room temperature. Membranes were then washed, incubated in secondary antibody and imaged (as above). Densitometric band analysis was performed with Quantity One 1-D Analysis Software (Bio-Rad, Hercules, CA, USA). Quantified protein expression values were normalized to β-actin.

### Data acquisition, statistical analyses and presentation

All data were collected at 1 kHz with the use of the PowerLab data acquisition system (AD Instruments, Colorado Springs, CO, USA) coupled to a computer. Heart rate (HR) and mean arterial pressure (MAP) were calculated from arterial blood pressure recordings. VC was calculated as a ratio of Q_fem_/MAP. For all conditions, Q_fem_, VC, MAP and HR were calculated as a 5 minute stable average during baseline (Baseline) and as a 1 minute average at the peak of drug response (Drug).

Statistical analyses were performed using Prism (version 4, GraphPad Software Inc, La Jolla, CA, USA) and differences were accepted as statistically significant when *p*<0.05. Effect of treatment on MAP and HR within each group was analyzed using a paired t-test, and between groups at baseline and drug using an unpaired t-test with a Bonferroni correction (2 comparisons; *p*<0.025). Effects of each drug perturbation on Q_fem_ and VC between CTRL and PD were analyzed using unpaired t-tests. The potential synergy between Y_1_R and α_1_R activation was assessed by comparing the sum of the drug responses from the BIBP3226 and prazosin conditions against those of the BIBP3226+prazosin condition using a paired t-test. Unpaired t-tests were used to compare cellular data (from Western blot and immunoassay) between groups. Pearson's Correlation was used to assess correlation between body mass and Q_fem_ or VC. Data are presented as mean values ± standard error (SE).

## Results

### Baseline

Body mass, blood glucose, insulin, lactate, mean end tidal CO_2_ and respiratory rate were significantly greater in PD *versus* CTRL (*p*<0.001, Table 1), however expired O_2_ and blood pH were similar between groups (Table 1). At baseline, both groups displayed similar MAP (85–95 mmHg), however HR was greater in PD *versus* CTRL (*p*<0.025, Table 2).

**Table 1:** Physical and physiological characteristics of CTRL and PD rats.

	CTRL	PD
Weight (g)	196±4	253±5*
Blood glucose (mmol/L)	9.3±0.6	14.1±0.9*
Insulin (nmol/L)	0.1±0.03	5.6±0.7*
Blood lactate (mmol/L)	1±0.1	2±0.1*
Expired CO_2_ (mmHg)	35±0.5	39±0.5*
Expired O_2_ (%)	17±0.1	17±0.1
Respiratory rate (breaths/min)	68±2	82±2*
Blood pH	7.4±0.01	7.4±0.01

Values are mean ± SE. CTRL, control, n = 7–8; PD, pre-diabetic, n = 7–9.

* *p*<0.001 vs. CTRL.

**Table 2:** Blood pressure and heart rate responses associated with each condition.

		BIBP3226		Prazosin		BIBP3226+Prazosin	
		CTRL	PD	CTRL	PD	CTRL	PD
Mean arterial pressure (mmHg)	Baseline	95±2	102±6	88±4	102±5	88±4	102±5
	Drug	89±4	94±8	72±5*	76±6*	71±3*	71±6*
Heart rate (beats/min)	Baseline	375±7	414±7†	371±6	409±8†	371±6	409±8†
	Drug	379±6	430±11* †	368±7	413±10†	368±8	406±7†

Values are mean ± SE. CTRL, control, n = 8; PD, pre-diabetic, n = 9.

* *p*<0.05 vs. Baseline.

† *p*<0.025 vs. CTRL.

Baseline Q_fem_ and VC were similar between groups and similar before each drug perturbation (Table 3). This observation was independent of body mass, as there was no correlation between body mass and Q_fem_ or VC (r = 0.11, *p* = 0.65). Vehicle infusion of saline had no effect on MAP, HR, Q_fem_ or VC in either group.

**Table 3:** Baseline values of hindlimb blood flow and vascular conductance before pharmacological treatments.

	BIBP3226		Prazosin		BIBP3226+Prazosin	
	CTRL	PD	CTRL	PD	CTRL	PD
Hindlimb blood flow (µl/min)	385±69	364±42	364±61	358±34	364±61	358±34
Vascular conductance (µl/min/mmHg)	4.0±0.6	3.7±0.5	4.1±0.6	3.6±0.4	4.1±0.6	3.6±0.4

Values are mean ± SE. CTRL, control, n = 8; PD, pre-diabetic, n = 9.

**Figure 1:**
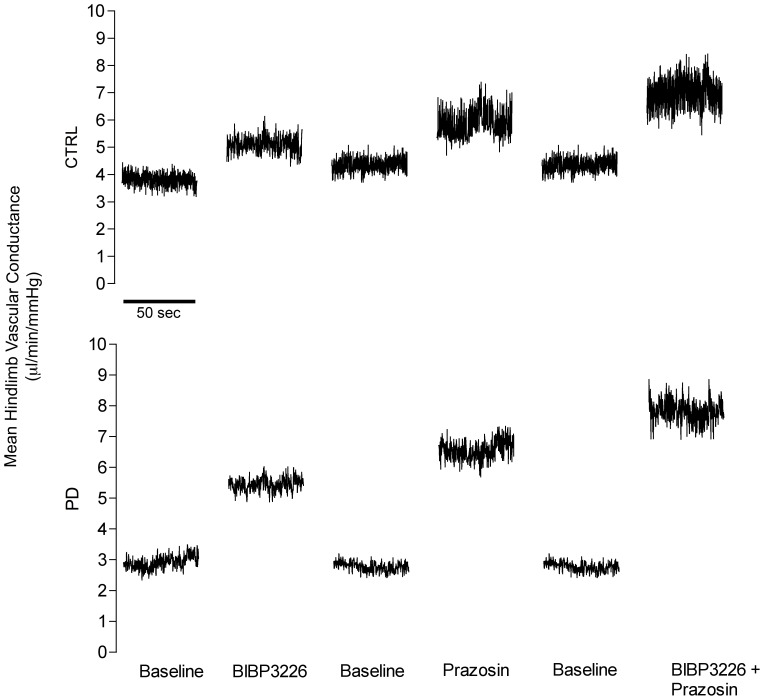
Representative mean vascular conductance (0.1 second averaging of 1 kHz beat-by-beat tracing) over 50 seconds for BIBP3226, prazosin and BIBP3226+prazosin treatments in CTRL (top) and PD (bottom).

The magnitude of vascular responses to bolus infusions of ACh and SNP were similar between groups, indicating that endothelial and smooth muscle cell functionality were preserved in PD (Table 4). Representative tracings of mean hindlimb vascular conductance to pharmacologic interventions are shown for a CTRL and PD rat in Figure 1.

**Table 4:** Hindlimb blood flow and vascular conductance at baseline and following acetylcholine and sodium nitroprusside interventions.

		Acetylcholine		Sodium Nitroprusside	
		CTRL	PD	CTRL	PD
Hindlimb blood flow					
(µl/min)	Baseline	380±50	395±30	385±66	377±47
	Drug	708±66*	760±93*	503±72*	582±53*
Vascular conductance (µl/min/mmHg)	Baseline	4.2±0.6	3.7±0.5	4.2±0.7	3.6±0.6
	Drug	10±1*	10±1*	12±2*	11±2*

Values are mean ± SE. CTRL, control, n = 6–8; PD, pre-diabetic, n = 6–8.

* *p*<0.05 vs. Baseline.

### Functional effects of local Y_1_R and α_1_R blockade

#### Effect of local Y_1_R blockade (BIBP3226)

**Figure 2:**
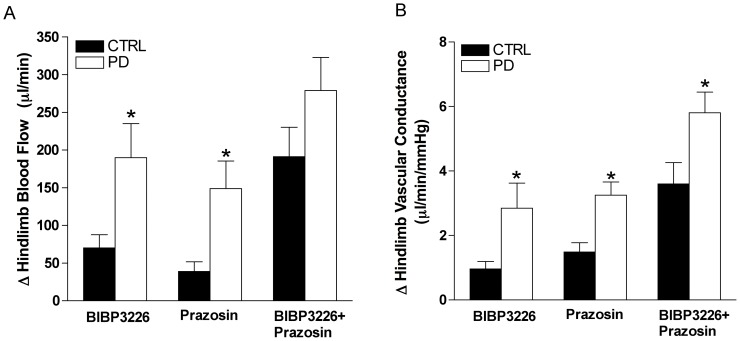
Sympathetic receptor blockade elicits greater vascular responses in PD. Panel A: Change in hindlimb blood flow (Q_fem_) and Panel B: vascular conductance (VC) from baseline following Y_1_R and α_1_R blockade. With Y_1_R blockade, the increase in Q_fem_ and VC was greater in PD (n = 9) *versus* CTRL (n = 8) (*p*<0.05). α_1_R blockade elicited an increase in Q_fem_, as well as an increase in VC that was greater in PD compared to CTRL (*p*<0.05). * Indicates different from CTRL (*p*<0.05).

**Figure 3:**
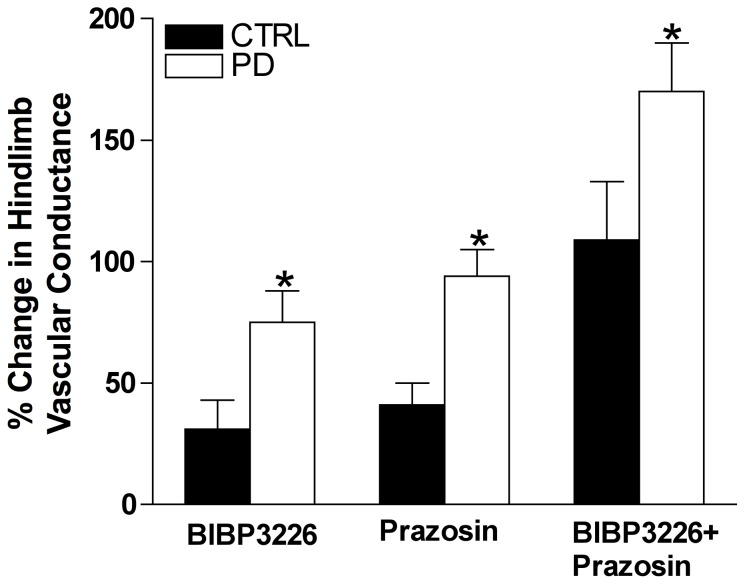
Percent change in hindlimb vascular conductance (VC) following Y_1_R and α_1_R blockade. The percent increase in VC following BIBP3226, prazosin and BIBP3226+prazosin treatments was greater in PD (n = 9) compared to CTRL (n = 8). *Indicates different from CTRL (*p*<0.05).

Following Y_1_R antagonism, MAP was unchanged for both groups, however HR increased from baseline in PD (*p*<0.05, Table 2). Q_fem_ and VC increased from baseline in CTRL (ΔQ_fem_ = 70±17 µl/min; ΔVC = 1.0±0.2 µl/min/mmHg^−1^) and PD (ΔQ_fem_ = 190±45 µl/min; ΔVC = 2.8±0.8 µl/min/mmHg) (*p*<0.05), however the increase in Q_fem_ and VC following Y_1_R blockade was greater in PD compared to CTRL (*p*<0.05, Figure 2). Percent change in VC was greater in PD (75±13%) *versus* CTRL (31±12%) (*p*<0.05, Figure 3).

#### Effect of local α_1_R blockade (prazosin)

Following α_1_R antagonism, MAP decreased 15±2 and 26±5 mmHg, for CTRL and PD respectively (*p*<0.05, Table 2) and HR was unchanged from baseline (Table 2). Q_fem_ and VC increased from baseline in CTRL (ΔQ_fem_ = 39±13 µl/min; ΔVC = 1.5±0.3 µl/min/mmHg) and PD (ΔQ_fem_ = 149±37 µl/min; ΔVC = 3.2±0.4 µl/min/mmHg), where the increase in Q_fem_ and VC was greater in PD compared to CTRL (*p*<0.05, Figure 2). Percent change in VC was greater in PD (94±11%) *versus* CTRL (41±9%) (*p*<0.05, Figure 3).

#### Effect of simultaneous Y_1_R and α_1_R blockade (BIBP3226+prazosin)

Following combined Y_1_R and α_1_R antagonism, MAP decreased 17±3 and 31±6 mmHg, for CTRL and PD respectively (*p*<0.05, Table 2), whereas HR remained unchanged. Q_fem_ and VC increased from baseline in CTRL (ΔQ_fem_ = 191±39 µl/min; ΔVC = 3.8±0.7 µl/min/mmHg) and PD (ΔQ_fem_ = 279±44 µl/min; ΔVC = 5.8±0.6 µl/min/mmHg) (*p*<0.05), however the increase in Q_fem_ and VC following combined Y_1_R and α_1_R blockade was greater in PD compared to CTRL (*p*<0.05, Figure 2). Percent change in VC was greater in PD (170±20%) *versus* CTRL (109±24%) (*p*<0.05, Figure 3).

**Figure 4:**
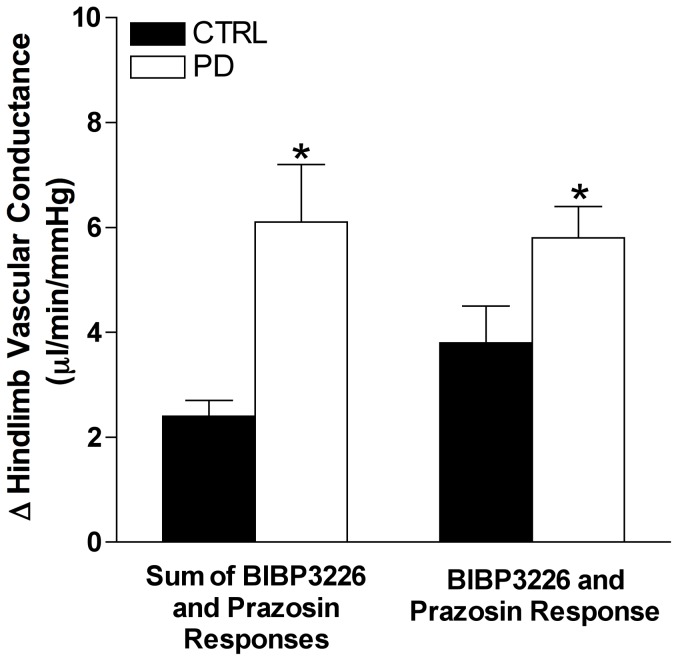
Y_1_R and α_1_R synergism is not observed in CTRL and PD. Comparison of the change in hindlimb vascular conductance between CTRL (n = 8) and PD (n = 9) for the sum of responses from BIBP3226 and prazosin conditions and the BIBP3226+prazosin condition. * Indicates different from CTRL (*p*<0.05).

To determine the potential synergistic interaction between endogenous Y_1_R and α_1_R activation, the sum of the VC responses from the BIBP3226 and prazosin conditions was compared to the VC responses elicited by combined Y_1_R and α_1_R blockade within each group. Compared to the sum of the independent effects of BIBP3226 and prazosin infusion, combined blockade resulted in a similar increase in VC within groups (Figure 4).

### Tissue NPY concentration and Y_1_R and α_1_R expression

#### Tissue NPY concentration

**Figure 5:**
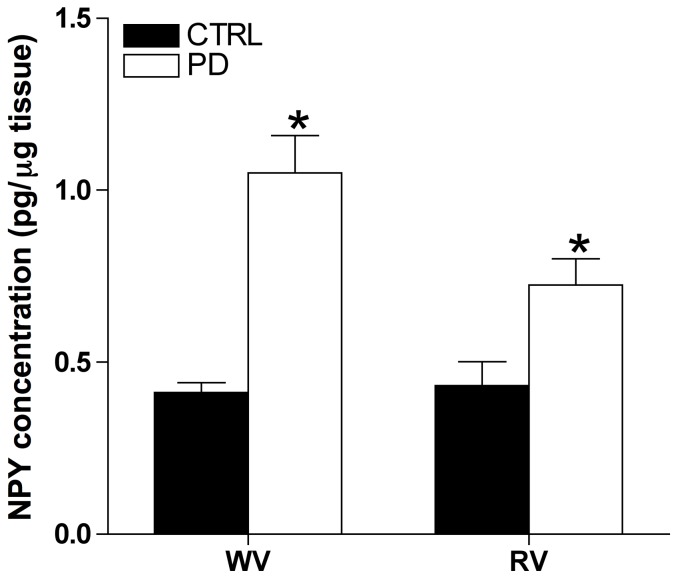
Skeletal muscle NPY concentration is elevated in PD. NPY concentration normalized to total protein for whole muscle homogenate of white vastus (WV) and red vastus (RV). PD (n = 6 per muscle group) tissue had greater NPY concentration compared to CTRL (n = 6 per muscle group). * Indicates different from CTRL (*p*<0.05).

NPY concentration was 155±32% and 68±32% greater in white and red vastus respectively in PD compared to CTRL (*p*<0.05, Figure 5).

#### Tissue Y_1_R and α_1_R protein expression

**Figure 6:**
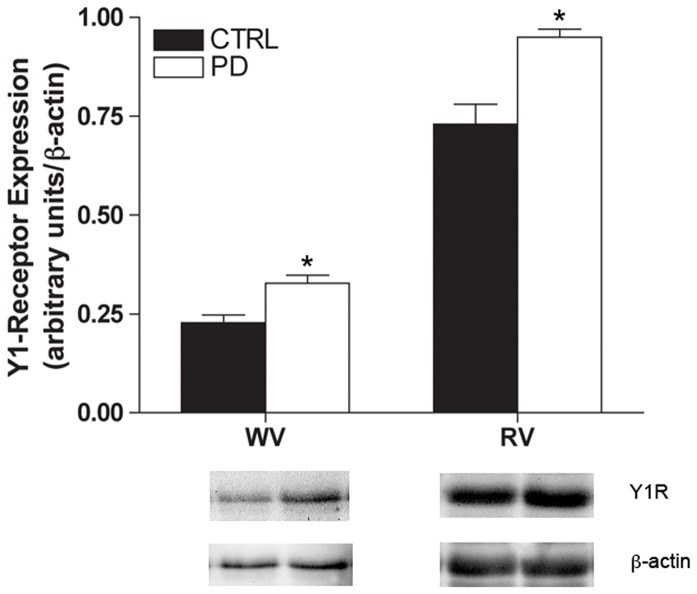
Y_1_R expression is augmented in PD. Western blot analysis of Y_1_R expression (∼43 kDa) in hindlimb muscle homogenate of CTRL (n = 6 per muscle group) and PD (n = 6 per muscle group). PD had greater overall expression of Y_1_R in both white and red vastus muscles. * Indicates different from CTRL (*p*<0.05).

**Figure 7:**
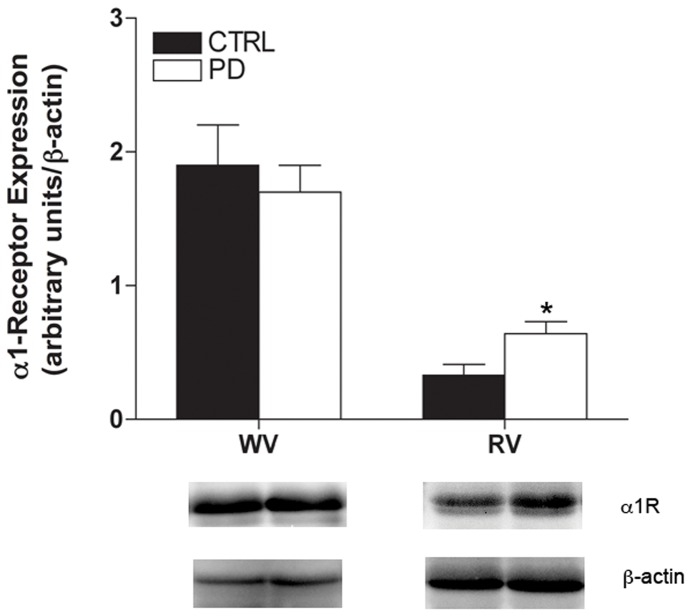
α_1_R expression is augmented in PD. Western blot analysis of α_1_R expression (∼42 kDa) in hindlimb muscle homogenate of CTRL (n = 6 per muscle group) and PD (n = 6 per muscle group). PD had greater α_1_R expression in red vastus muscle, compared to CTRL. * Indicates different from CTRL (*p*<0.05).

Compared to CTRL, Y_1_R protein expression was 43±15% and 30±9% greater in PD white and red vastus muscle respectively (*p*<0.05, Figure 6). α_1_R expression was 94±43% greater in PD compared to CTRL in red vastus muscle (*p*<0.05), however expression in white vastus muscle was similar between groups (Figure 7).

## Discussion

As hypothesized, we observed heightened sympathetic influences on baseline vascular control in pre-diabetes, as blockade of sympathetic receptors elicited greater Q_fem_ and VC responses in PD compared to CTRL. This is the first study to report that pre-diabetes promotes an overall increase in Y_1_R and α_1_R vascular control under baseline conditions. Accordingly, increases in skeletal muscle NPY concentration and Y_1_R expression were observed in PD. However, in contrast to our hypothesis, we did not unmask Y_1_R and α_1_R synergistic effects on VC with combined receptor blockade.

In the current study we demonstrated that modifications in sympathetic vascular control occur before the manifestation of endothelial and/or vascular smooth muscle dysfunction generally observed in overt type 2 diabetes. Our current data are supported by past studies (using the same model of pre-diabetes) showing no differences in responses to ACh or SNP in PD versus CTRL [20], [33]. These data indicate that endothelium dependent and independent responses to such pharmacological stimuli (ACh and SNP) are intact in PD, supporting the hypothesis that vascular dysregulation in early pre-diabetes is mainly due to modifications in sympathetic control.

Past work from our group suggests that sympathetic vascular control involves interactions between Y_1_R and α_1_R [27]. Until presently, there was a lack of research investigating the role of NPY in pre-diabetic vascular dysfunction. In fact, past investigations addressing augmented sympathetic vascular control in pre-diabetes have relied predominantly on the functional responses to infusion/application of α-adrenergic agonists *in vivo*, or responses of isolated vascular preparations treated with these agents [20], [34]. Although essential for determining the existence of receptors and their independent function(s) within physiological systems, the infusion of agonists does not address autogenous ligand–receptor interactions. In the current investigation highly selective Y_1_R and α_1_R antagonists (BIBP3226 and prazosin respectively) were delivered alone and in combination to address endogenous independent and synergistic Y_1_R/α_1_R control under baseline conditions. Although responses to Y_1_R, α_1_R, and combined blockade were markedly augmented in PD, we did not unmask endogenous Y_1_R and α_1_R synergism in either CTRL or PD (Figure 3). This was surprising, as we have previously reported endogenous synergy between Y_1_R and α_1_R in adult male Sprague Dawley rats [27]. Thus, it seems that such receptor interactions are not present in the young ZDF rat or they were not robust enough to resolve in the current study.

Despite similar baseline Q_fem_ and VC among groups, we observed that both Y_1_R and α_1_R sympathetic antagonist treatments resulted in greater vascular responses in PD. Under conditions of heightened sympathetic influence, it seems unexpected that similarities in baseline Q_fem_ and VC would exist. However, our observations are supported by other work where isolated vessels from pre-diabetic rats (with similar baseline tone) demonstrated greater responses to sympathetic agonists compared to controls [20]. Thus, in the current study, it appears that compensatory dilatory mechanisms served to maintain normal blood flow under baseline conditions in PD. The presence of high blood lactate (a potent vasodilator [35]) in PD likely contributed to buffering the effects of augmented sympathetic vascular modulation. In support of our data, others have shown that insulin resistance [36] and type 2 diabetes [37] are associated with heightened lactate levels.

Our observations of augmented baseline Y_1_R and α_1_R activation in PD are complemented by our findings that PD had greater NPY concentration and Y_1_R and α_1_R expression in hindlimb skeletal muscle. Neuropeptide Y is produced in sympathetic neuronal cell soma and packaged into secretory large dense-cored vesicles and undergoes axonal transport (the rate of which is SNA level dependent) to the axon terminal where it is released and eventually degraded by enzymes in the synaptic cleft [38]. This is in contrast to NE, which is produced in sympathetic nerve terminal, released, and eventually taken back up into the nerve terminal [39]. Based on the unique origin and fate of NPY, it can be reasonably inferred that increased skeletal muscle NPY concentration measured in PD was a result of one or a combination of the following: i) augmented sympathetic neuronal density; ii) increased production and axonal transport of NPY; and/or iii) increased NPY release into skeletal muscle interstitium. This line of reasoning falls in line with work by others who reported sympathetic nerve hyperactivity in insulin resistant and type 2 diabetic subjects, as well as heightened plasma NPY levels in type 2 diabetic patients [40], [41]. Beyond this, *in vivo* studies investigating NPY levels and Y_1_R/α_1_R expression in pre-diabetes are limited, however increased Y_1_R mRNA expression has been reported in cardiac tissue of diabetic rats [42] and it was shown that rat vascular smooth muscle cells treated with high levels of insulin resulted in upregulation of α_1_R [43].

### Limitations

We used hindlimb muscle homogenate in order to quantify the receptors located along downstream resistance arterioles, as these vessels are responsible for modulating flow at the level of the femoral artery. Previous work indicates that peripheral Y_1_Rs are predominantly associated with vasculature [44]. In contrast, α_1_Rs have been identified on skeletal muscle fibers in rats, however the density of those located in muscle fibers is negligible compared to α_1_R expression on resistance arterioles [45]. Based on past reports and the internal consistency between our functional and cellular data, we are confident that our reported differences in ligand concentration and receptor expression reasonably reflect what is occurring at the level of the vasculature.

We measured skeletal muscle tissue NPY concentration instead of plasma NPY levels for several reasons. Indeed, repeated blood sampling poses the risk of evoking hypotension and increases in sympathetic nerve activity. As well, plasma NPY levels represent a mixed sample originating from several sources throughout the body. In contrast, the skeletal muscle samples used in this study were promptly harvested from anesthetized animals (with minimal hemodynamic stress) under the same conditions that functional data were acquired. Thus, we feel that our reported NPY levels are an accurate representation of the local skeletal muscle environment under baseline conditions.

Due to limitations in detection, NE levels were not measured in the current study. However, this investigation and previous from our group [26], [27] used a sensitive enzyme immunoassay optimized to detect NPY in skeletal muscle homogenates. NPY is co-released and co-stored with NE [4] and plasma NPY release correlates with NE release [46], especially under conditions of elevated sympathetic nerve activity; thus, it is reasonable to postulate that our measures of increased skeletal muscle NPY concentration in PD reflect a concomitant increase in skeletal muscle NE.

In conclusion, we provide the first report that Y_1_R and α_1_R vascular regulation is augmented in the hindlimb of pre-diabetic ZDF rats. Our findings are supported by increased skeletal muscle NPY concentration and Y_1_R/α_1_R expression in PD *versus* CTRL. Future studies are required to ascertain the long-term cardiovascular consequences of our findings and their functional significance in contracting skeletal muscle.
